# Effectiveness of Trained Community Lay Workers on Glycemic Control, Knowledge, and Self-Efficacy Among Agricultural Workers with Diabetes in the Texas Panhandle

**DOI:** 10.1007/s10903-024-01603-8

**Published:** 2024-05-03

**Authors:** Anabel Rodriguez, Lus Chavez, Teresa Wagner, Carol Howe

**Affiliations:** 1 School of Public Health College Station, Texas A&M University, Environmental and Occupational Health, 212 Adriance Lab Road, 1266 TAMU, College Station, TX 77843 USA; 2 Family Support Services of Amarillo, Sembrando el Sueño, 2209 SW 7th, Amarillo, TX 79106 USA; 3 School of Health Professions, The University of North Texas Health Science Center at Fort Worth, 3500 Camp Bowie Blvd, IREB 470C, Fort Worth, TX 76107 USA; 4 Harris College of Nursing and Health Sciences, Texas Christian University, 2800 South University Drive, Fort Worth, TX 76109 USA

**Keywords:** Training, Diabetes, Rural health, Hispanic, Guatemala, K’iche’

## Abstract

Systemic health barriers, economic challenges, and lack of follow-up care exacerbate self-management of chronic diseases like diabetes among Hispanic agricultural workers. The primary objective of this pilot project was to determine the benefit of using community coaches to decrease A1C levels and increase diabetes knowledge among agricultural workers with diagnosed diabetes in the Texas Panhandle. A longitudinal study design with two phases was used to create, deliver, and evaluate a diabetes coaching program. Phase 1 was the development of the program and community coaches training (*n* = 4). In Phase 2, the coaches then delivered the program over 12 weeks to thirteen clients. *Phase 1*: All coaches were Hispanic females, 28.3 (SD 3.8) years of age, half had at most a high school education level and the other half had a vocational certification (*n* = 4). Mean DKQ-24 score was 54.2% (SD = 29.7) at baseline and 75.0% (SD = 31.4) after training (*t* (4) = 4.6, *P* < 0.05). We observed a very large difference between mean baseline and exit DKQ-24 scores relative to the pooled standard deviation, resulting in an effect size estimate of 0.59 indicative of a medium to large learning effect. *Phase 2*: Clients were Hispanic Spanish-speaking, predominantly female (55%), 44.4 (SD 6.8) years of age with at most a high school level of education (88.9%) and occupations varied from dairy farm worker (33.3%), meat processing worker (33.3%), and other agriculture or manufacturing position (33.3%). The mean SKILLD score was 40.0% (SD = 28.7) at baseline, increasing to 72.2% (SD = 25.4) at 12 weeks upon completion of the coaching program (*t* (9) = 2.956, *P* < 0.05). We observed a very large difference between mean baseline and exit SKILLD scores relative to the pooled standard deviation, resulting in an effect size estimate of 1.13 indicative of a large learning effect. The mean A1C levels at baseline screening was 8.3% (SD = 3.0) and 7.6% (SD = 3.0) at exit screening, representing a 0.7% decrease (*p* = 0.4730). No statistically significant differences were observed between depression (*p* = 0.786) or anxiety (*p* = 1.000) measures at baseline compared to exit. Training and coaching programs for hard-to-reach agricultural and meat processing workers must be culturally, linguistically, and literacy appropriate for both coaches and clients. The program must be feasible and sustainable, focus on empowering community members, capitalize on technological advances and persisting new-normals from the COVID-19 pandemic as well as dismantle common systemic barriers to health and understanding lived-experiences of agricultural working populations in rural regions.

## Introduction

A healthy and present workforce is crucial for the sustainability of modern agricultural production in the United States (U.S.) [1]. U.S. agricultural workers are predominantly immigrant [2], Hispanic males [3], of approximately 30 years of age [4] with limited English proficiency and formal education [5]. Most agricultural workers are of Mexican descent (88.5-97.1%) [4–6]. In Texas and New Mexico, there has been an increase in agricultural workers originating from the department of Quiché in Guatemala [7, 8]. In the Texas Panhandle, most immigrants find occupations deemed low-skill with low wages in the prominent dairy, farming, animal husbandry, and meatpacking industries, with little to no social support [9]. 

Diabetes (12.5%) among U.S. Hispanics is highest compared to non-Hispanic Blacks, Asians, and Whites (7.5%) [10] with nearly 40.0% of agricultural workers unaware of their diabetes status [11]. Rural agricultural workers often have limited access to healthcare services [12]. Barriers to access include cost, transportation, language challenges, absence of health insurance, cultural differences, limited knowledge of health centers and locations, time conflicts due to work schedules, lack of childcare, unavailability of specialty services, migratory lifestyles, and fear of law and immigration enforcement [11–21]. This can lead to the progression of diabetes with increased disease severity [13]. Consequently, increased severity of chronic illnesses leads to increased presenteeism (physically present at work but sick, stressed, or tired) [22] and absenteeism (time absent from work) [23] which leads to lost wages, decreased access to health due to costs, and ultimately lower quality of life.

One proposed way to decrease A1C levels and increase the self-management and self-efficacy among patients with diabetes is to harness social and peer support of culturally and linguistically matched community lay workers [24, 25]. The true burden of the lack of diabetes knowledge, management, and self-efficacy among Mexican (Spanish) and Guatemalan (K’iche’) agricultural workers with diabetes in the Texas Panhandle remains largely unknown [26–28]. There is a need to determine the benefits of using community lay coaches to improve A1C and knowledge about diabetes among agricultural workers. These data may inform data-driven decisions to increase the use of community coaches to help medically underserved rural communities. The primary objective of this pilot project was to determine the benefit of using community coaches to decrease A1C levels and increase diabetes knowledge among agricultural workers with diabetes in the Texas Panhandle.

## Methods

A longitudinal study design with two phases was used to create, deliver, and evaluate a diabetes coaching program. Phase 1 was the program development and community coaches training. In Phase 2, the coaches delivered the program to clients over 12-weeks. This study was approved by the University of Texas Health Science Center at Houston Committee of the Protection of Human Subjects (HSC-SPH-21-0740).

### Phase 1: Community Coaches

#### Participants

Eligibility criteria for coaches included being over age 18, speaking or understanding English and Spanish and/or Spanish and K’iche’. Coaches were invited from a list of community members from the Cactus Nazarene Ministry Center (CNMC), dedicated to addressing immigration, children programming, and healthcare needs of the diverse community of Cactus, Texas. Four linguistically diverse community members from or living near Moore County were recruited to participate in this training. After receiving information about the study, all signed an electronic consent on Qualtrics Mobile Survey Software®.

### Diabetes Education Training

**Fig. 1:**
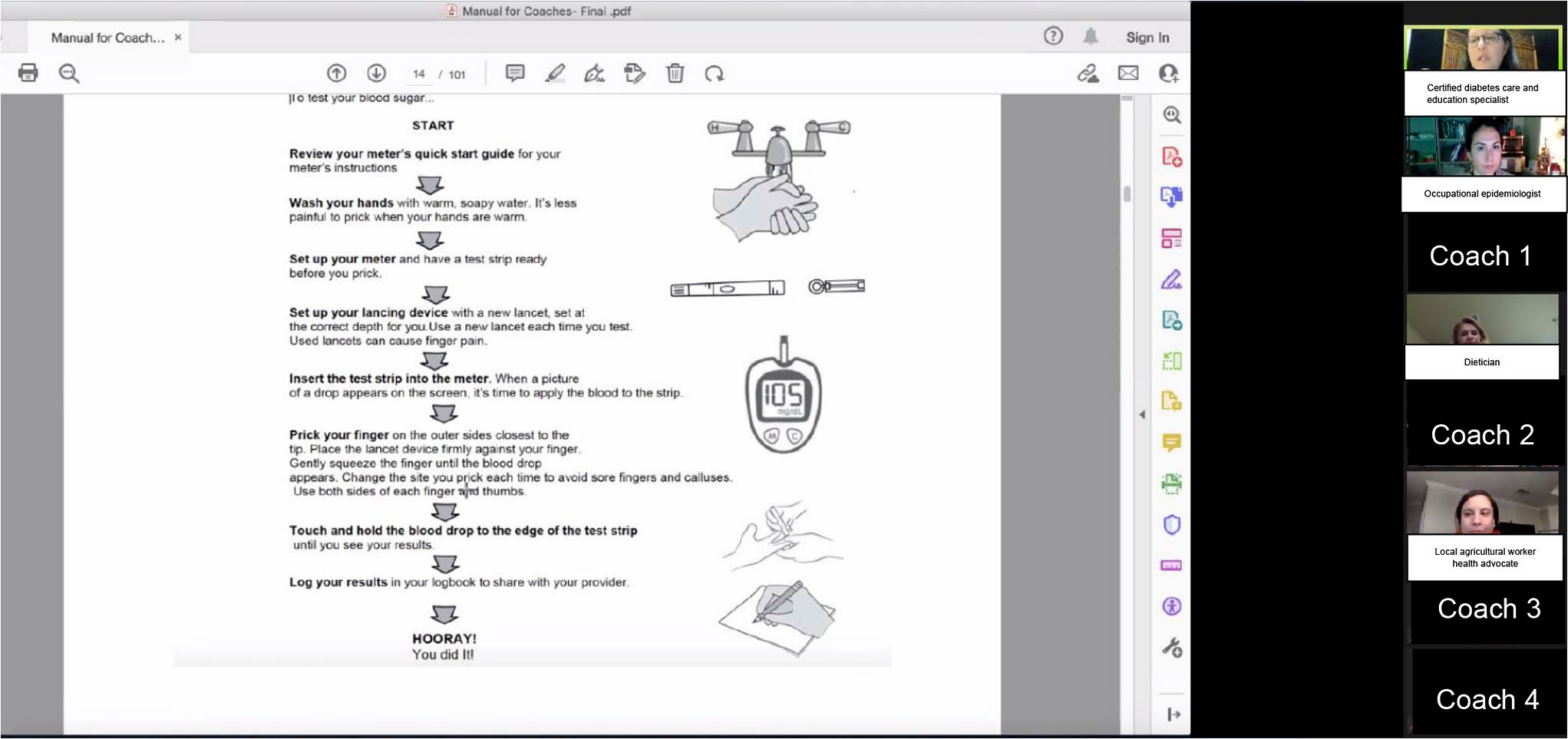
Coaches 5-week virtual training. Registered nurse and certified diabetes care and education specialist demonstrating how to properly use a lancing device during glucometer use module. *Coaches identities hidden for confidentiality purposes*

Research personnel included a registered nurse and certified diabetes care and education specialist, dietician, occupational epidemiologist, and a local agricultural worker health advocate. The diabetes care nurse and dietician developed the diabetes training program focused on (1) community coach role (2) what is diabetes, (3) healthy eating, (3) blood glucose levels and monitoring, (4) diabetes medicine, (5) physical activity, (6) early identification and treatment of complications, (7) coping with stress and depression, (8) goal setting (Table 1) [24]. A curriculum manual was provided to guide their weekly coaching sessions. Research personnel delivered the training program in English in 1-hour sessions over five-weeks, using the manual for visuals, real-life examples, and role-playing exercises (Fig. 1). The training modeled the coaching that coaches would do with participating agricultural workers with diabetes (referred to as ‘their clients’). The research team reviewed surveys that the coaches would complete with their clients. In-person training was scheduled to begin January 2022; however, COVID-19 spikes and health and safety institutional guidelines pushed us to engage virtually. One research team member acted as the local contact to manage study logistics and support coaches. Coaches received a signed certificate and $1,000 in e-gift cards over 6-months.

**Table 1:** Community health worker diabetes education curriculum

Session	Topic	Learning Objective
1	Peer leader role What is diabetes	Describe what diabetes is Demonstrate how to monitor blood sugar with meter Identify target blood sugar Describe symptoms and treatment high blood sugar
2	Healthy eating	Identify foods (carbohydrates) that make blood sugar go up Understand MyPlate for portions Set goal for healthy eating
3	Physical activity	Understand physical activity helps to decrease blood sugar List positive effects of regular physical activity Set goal for physical activity
4	Stress, coping	Describe how stress increases blood sugar Identify healthy coping strategies Identify resources for coping
5	Teaching and support practice	Role play to provide teaching on a topic Provide feedback to each peer

### Measures

General diabetes knowledge was assessed at baseline and five-weeks upon completion of the diabetes training program with the reliable and validated 24-item version of the Diabetes Knowledge Questionnaire (DKQ-24) [28]. DKQ-24 questions assess knowledge of medications, diet, exercise, home glucose monitoring, foot care, and treatment modifications. Each item can be answered as ‘Yes,’ ‘No,’ or ‘I don’t know.’ Each answer is weighted equally, scored as 1 if ‘correct’ and 0 if ‘incorrect’, and totaled to maximum score of 24 points.

### Phase 2. Agricultural Workers with Diabetes

#### Study Design

A pre-post test design was used to assess agricultural workers’ diabetes knowledge, A1C, depression, anxiety, before and after individualized coaching from trained community coaches.

### Setting

The Well Health Center is a faith-based charitable clinic that provides health care to the immigrant and refugee community in Moore County. A clinic needs assessment found that one-third of patient encounters were for diabetes care with over 60% being Hispanic (Mexican and Guatemalan) patients. Clinic leadership and staff were key in collaborating and providing patient data, meeting space, A1C screening services, and medical staff support. Coaching and data collection took place between March and June 2022.

### Sample

No power analysis for sample size was warranted because this exploratory study was hypotheses generating. Sample size was deliberated with The Well Health Center medical staff and determined based on time, cost, and feasibility. Eligibility criteria included being ≥ 18 years of age, speaking Spanish, K’iche’, or English, working in the agricultural industry, and having diabetes. Interested and eligible agricultural workers were scheduled for a meeting at the clinic. A total of 13 agricultural workers (10 Spanish and 3 K’iche’-speaking) were initially recruited. After local research personnel read and explained the study, clients signed an electronic informed consent on Qualtrics Mobile Survey Software®.

### Coach-Client Assignments

Trained community coaches and clients were matched based on language and cultural similarities and were introduced in-person at the clinic by the local research personnel. Coaches and their clients exchanged contact information and arranged for follow up coaching sessions.

### Coach-Client Follow-up

Clients were coached weekly on diabetes topics using the 12-week manual with the learning objectives and curriculum outlined for each week. Local research personnel served as support and followed-up with coaches via phone and in-person on a weekly basis. In case of specific medical questions, coaches were advised to contact study investigators and/or clinic staff.

### Measures

Sociodemographic information of clients was collected, including gender, age, nationality, language, education-level, marital status, and occupation. Coaches completed all surveys verbally with patients. Client A1C levels were measured at The Well Health Center. A decrease of 0.5% in A1C levels was considered clinically significant [29]. 

Diabetes knowledge was assessed with the validated 10-item Spoken Knowledge in Low Literacy patients with Diabetes (SKILLD) [27]. SKILLD questions assessed knowledge of hyperglycemia, hypoglycemia, foot care, eye exams, fasting glucose, A1C, exercise, and long-term complications of diabetes. Each item is weighed equally at 10 points for a total of 100 points. A total score > 50% indicates ‘high knowledge’ whereas ≤ 50% indicates ‘low knowledge.’

Depression was measured using the well validated Patient Health Questionnaire-9 (PHQ-9) [30]. Each of the 9 items were scored from 0 (not at all) to 3 (nearly every day). Total scores and depression categories include: 0–4 as none, 5–9 as mild, 10–14 as moderate, 15–19 as moderately severe, and > 20 as severe [31, 32]. 

Anxiety was measured using the General Anxiety Disorder-7 (GAD-7), a seven-item, self-report questionnaire to assess general anxiety during the previous 2 weeks. Patients rate the 7 items from 0 (not at all) to 3 (nearly every day). Total scores and anxiety categories include: 0–4 as minimal anxiety, 5–9 as mild anxiety, 10–14 as moderate anxiety, and > 15 as severe anxiety [33]. 

**Fig. 2:**
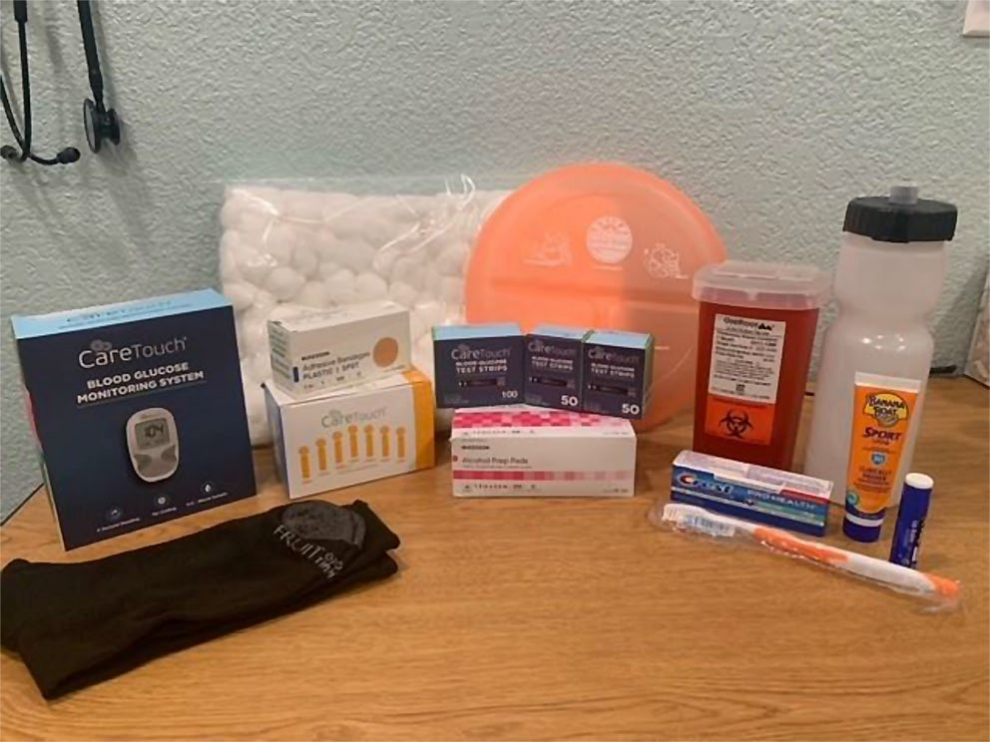
Diabetes kit used by coaches during 12-week training program: glucometer, lancets, strips, alcohol prep pads, adhesive bandages, cotton balls, biohazard container for lancets, portion control plate, water bottle, toothbrush, toothpaste, lip balm, sunscreen and work boot socks

SKILLD, PHQ-9, and GAD-7 measurements were collected at baseline and upon completion of the coaching program. Participants were compensated with a total of $100 in e-gift cards and a diabetes care-kit (Fig. 2).

### Data Analyses

Statistical analysis included descriptive statistics of sociodemographic characteristics, test score results, and training effectiveness evaluations. Paired *t-test* were used to assess knowledge improvement for coaches on DKQ-24 and patient’s SKILLD and A1C at baseline and exit. Hedges’ *g* estimate was used to compute effect size based on a comparison of baseline and exit DKQ-24 and SKILLD mean scores relative to pooled variances. Fisher’s exact test (with one or more cells < 5) was used to measure relationships between categorical variables PHQ-9, GAD-7, and SKILLD (low and high knowledge categories) at baseline and exit. All statistical analyses was performed using Stata SE 17.0 software [34]. 

## Results

### Results Phase 1: Coaches

#### Sociodemographic Characteristics

Coaches were all female, Hispanic, with a mean age of 28 years and most were born in the U.S. and English was their primary language. Half had high school education and the other half vocational certifications. Half were married and all had children. Coaches worked in nursing or childcare and worked fulltime (Table 2).

**Table 2:** Sociodemographic characteristics of diabetes program coaches (*n = 4*)

Characteristics	All (*n* = 4)
	Mean (SD) or *n* (%)
Age (*Range 24–33*)	28.3 (3.8)
Female	4 (100.0)
*Nationality*	
United States	3 (75.0)
Guatemala	1 (25.0)
Hispanic	4 (100.0)
*Primary language*	
English	3 (75.0)
K’iche’	1 (25.0)
*Secondary language*	
Spanish	4 (100.0)
*Education*	
High school	2 (50.0)
Vocational/College	2 (50.0)
*Marital status*	
Single	2 (50.0)
Married	2 (50.0)
Children	4 (100.0)
Number of children (*Range 1–5*)	2.8 (2.1)
*Full-time occupation*	
Vocational nurse	2 (50.0)
Childcare provider	1 (25.0)
Self-employed	1 (25.0)
Days worked per week (*Range 3–6*)	4.8 (1.3)
Hours worked per day (*Range 8–10*)	8.8 (1.0)

### Baseline and Exit Evaluation—DKQ-24

Mean DKQ-24 score increased from 54.2% (SD = 29.7) at baseline to 75.0% (SD = 31.4) after training (*t* (4) = 4.6, *P* < 0.05). We observed a very large difference between mean baseline and exit DKQ-24 scores relative to the pooled standard deviation, resulting in an effect size estimate of 0.59 indicative of a medium to large learning effect (Table 3).

**Table 3:** Coach results for 24-item Diabetes Knowledge Questionnaire (DKQ-24) by education, country of origin, and primary language (*n = 4*)

	Baseline (SD) (*n = 4*)	Exit (SD) (*n = 4*)
All participants*	54.2 (29.7)	75.0 (31.4)
*Highest education level achieved*		
High school	41.7 (29.2)	56.3 (27.1)
Vocational or College	66.7 (12.5)	93.8 (6.3)
*Country of origin*		
United States	68.1 (90.3)	90.3 (5.0)
Guatemala	12.5 (0.0)	29.2 (0.0)
*Primary language*		
English	68.1 (90.3)	90.3 (5.0)
K’iche’	12.5 (0.0)	29.2 (0.0)

* Statistically significant (*p* < 0.05) differences between DKQ-24 baseline and exit scores

### Results Phase 2: Clients

#### Sociodemographic Characteristics

Mean age of clients was 44.4 (SD 6.8, Range 37–56) and 55% identified as female (Table 4). All were Spanish-speaking Hispanic, with most from Mexico, and had lived in the U.S. for over 20 years. Most had a high school education, were married, and had children. All worked in agriculture or meat processing, working 5 days a week for 9 h per day. Most had been living with diabetes for an average of 5 years and almost all were vaccinated for COVID-19. About 1/3rd reported Facebook and WhatsApp as the most trusted social media platforms for health information.

**Table 4:** Sociodemographic characteristics of agricultural workers with diabetes (*n = 9*)

Characteristics	All (*n* = 9)
	Mean (SD) or *n* (%)
Age (*Range 37–56*)	44.4 (6.8)
Female	4 (50.0)
*Nationality*	
Mexico	8 (88.9)
Guatemala	1 (11.1)
Years in U.S. (*Range 5–40*)	23.8 (10.8)
Hispanic	9 (100.0)
*Primary language*	
Spanish	9 (100.0)
*Education*	
Less than high school	8 (88.9)
College or higher	1 (11.1)
*Marital status*	
Married	5 (55.6)
Children	7 (77.8)
Number of children (*Range 2–5*)	3.5 (1.0)
*Occupation*	
Dairy farm	3 (33.3)
Meat processing	3 (33.3)
Other agriculture or manufacturing	3 (33.3)
Years of experience (*Range 5–14*)	10.5 (3.3)
Days worked per week (*Range 3–7*)	5.3 (1.5)
Hours worked per day (*Range 4–12*)	9.2 (2.7)
*Trusted social media for health information*	
Facebook	3 (33.3)
WhatsApp	3 (33.3)
Instagram	1 (11.1)
None	2 (22.2)
Years diagnosed with diabetes (*Range 1–23*)	5.3 (7.5)
COVID-19 vaccinated	8 (88.9)

### Baseline and Exit Evaluation—Skilld and A1C

The mean SKILLD score was 40.0% (SD = 28.7) at baseline, increasing to 72.2% (SD = 25.4) at 12-weeks upon completion of the coaching program (*t* (9) = 2.956, *P* < 0.05). We observed a very large difference between mean baseline and exit scores relative to the pooled standard deviation, resulting in an effect size estimate of 1.13 indicative of a large learning effect. The mean A1C baseline level was 8.3% (SD = 3.0) decreasing to 7.6% (SD = 3.0) at exit screening, representing a 0.7% decrease (*p* = 0.4730). The goal for adults with diabetes is an A1C < 7, and changes in A1C greater than 0.5% in response to interventions is considered clinically significant [29] given the strong negative association between A1C and risk of complications, including nephropathy, retinopathy, and cardiac events [35]. 

### Depression and Anxiety

At baseline, the majority of clients experienced from none to mild depression with one participant experiencing severe depression (Table 5). At program exit, participants experienced from none to moderate depression, with no participants experiencing severe depression. No statistically significant difference was observed between depression measures at baseline compared to exit (*p* = 0.786)—but at least one client improved from severe depression to moderate depression. At baseline, participants experienced minimal to moderate anxiety—with no participants experiencing severe anxiety. At program exit, participants experienced minimal to mild anxiety, with no participants experiencing moderate or severe anxiety. No statistically significant differences were observed between anxiety measures at baseline compared to exit (*p* = 1.000).

**Table 5:** Program baseline and exit evaluation measures for agricultural workers with diabetes (*n* = 9)

	Baseline (*n = 8*)	Exit (*n = 9*)	p*
	Mean (SD) or *n* (%)		
*Depression measure (PHQ-9)*			
None (0–4)	3 (37.5)	2 (22.2)	0.786
Mild (5–9)	3 (37.5)	5 (55.6)	
Moderate (10–14)	1 (12.5)	2 (22.2)	
Moderately severe (15–19)	-	-	
Severe (20–27)	1 (12.5)	-	
*Anxiety measure (GAD-7)*			
Minimal anxiety (0–4)	5 (55.6)	6 (66.7)	1.000
Mild anxiety (5–9)	3 (33.3)	3 (33.3)	
Moderate anxiety (10–14)	1 (11.1)	-	
Severe anxiety (> 15)	-	-	
*SKILLD*	40 (28.7)	72.2 (25.4)	0.018*
≤50% (low knowledge)	6 (66.7)	2 (22.2)	0.583
>50% (high knowledge)	3 (33.3)	7 (77.8)	
A1C clinical measure (*Range 6.2–15.5*)	8.3 (3.0)	7.6 (3.0)	0.473

* Statistically significant (*p* < 0.05) differences between baseline and exit evaluations

## Discussion

Clients experienced a decrease in mean A1C from 8.3 to 7.6%, a 0.7% decrease. Although this was not a statistically significant decrease in A1C levels post coaching program, a decrease > 0.5% is considered clinically significant [36]. The target for adults with diabetes is an A1C < 7%. However, A1C levels reflect an 8 to 12-week blood glucose average, taking time and consistent lifestyle changes to see noticeable decreases. A previous promotora-driven intervention for glycemic self-management among 70 farmworkers in the US-Mexico border saw decreased A1C levels by 1% among clients with high risk for diabetes complications in a 12-month period [25]. A larger sample size and a 12-month timeline was not feasible for this pilot project, but should be considered when planning interventions aimed at decreasing A1C levels among hard-to-reach populations.

Overall, clients increased their knowledge of diabetes on hyperglycemia, hypoglycemia, eye exams, fasting glucose level, A1C levels, exercise, and long-term complications of diabetes. The SKILLD evaluation was administered verbally by coaches in anticipation of low literacy and levels of education among participants [27]. Consistent with previous work in this region [7, 8, 37, 38], the majority of participants had less than a high school level education from Mexico or Guatemala. The SKILLD also allowed for participants to express logic behind answers provided compared to restrictive multiple-choice formatted questions. This recorded logic then allowed research personnel to determine correct and incorrect answers based on *Rothman et al.* rubric [27]. The SKILLD evaluation proved to be appropriate and feasible for agricultural workers with low literacy levels.

Findings from this pilot can guide the use of lay coaches in a larger multi-year study among agricultural workers with diabetes in rural health deserts similar to Moore County. Delivering and modelling the diabetes curriculum during training sessions that coaches in turn delivered to their clients was effective, confirming previous efforts. For example, a pilot program to reduce depression and stress among immigrant Latinas used community promotoras to conduct outreach to their ‘compañeras’ (local social network). The curriculum taught promotoras specific coping skills they then taught their compañeras to decrease perceived stress and depression [39]. 

Virtual delivery of the training program allowed for a rich cross-institutional collaboration and, simultaneously, a long-distance reach into a rural community with limited training resources. Virtual delivery granted coaches accessibility and flexibility. All four coaches successfully completed the 5-week training. Overall, the coaches training proved effective in knowledge gained. Worth noting, our Spanish – K’iche’ speaking coach was assigned to our local research member who would visit weekly to review training content. Despite additional time and resources allocated, our Spanish – K’iche’ speaking coach was out-performed by our English – Spanish speaking coaches. Potentially, this alludes to linguistic, cultural, and educational opportunity differences between Mexican Spanish and Guatemalan Spanish and K’iche’. Guatemalan K’iche’ only recently became a written language, and certain medical terms like ‘diabetes,’ ‘kidney failure,’ ‘insulin,’ are not part of Mayan vocabulary [40]. Future work should focus on creating and evaluating diabetes educational content in K’iche’ with guidance from the Guatemalan K’iche’ community and consideration of regional dialects. Content can include colorful illustrations and minimal text or animated videos with K’iche’ voice-over. Mobile technologies should also be considered as a delivery method to increase diabetes care knowledge among agricultural workers as previously proved effective in increasing safety awareness among Mexican and Guatemalan dairy farm workers in the Texas Panhandle by Rodriguez et al. [7]. In addition, trusted social media platforms like Facebook and WhatsApp should be leveraged to deliver health information to agricultural workers in rural and medically underserved regions.

In-person and ‘personal’ delivery of the coaching program was successful due to coaches being *embedded* in the community and being familiar with agricultural worker lifestyles. Methods for this second phase of the pilot project aimed at dismantling systemic barriers to healthcare access such as cost (program was free), transportation (coaches and participants lived in the same community), language and translation challenges (matched based on language), absence of health insurance (charitable clinic), cultural differences (matched based on culture), limited knowledge of health centers and locations (list of local low cost services provided), time conflicts due to demanding work schedules and lack of childcare (visits or calls around their working and family schedules), migratory lifestyles (contact via phones, WhatsApp), and fear of law and immigration enforcement (documentation status not asked and no travel required) [11–21]. Future research involving agricultural workers in rural regions should intentionally consider and address all barriers to access when planning all stages of programs. Simply neglecting to address one barrier can impact participation and study attrition.

## Limitations

Study limitations included small sample size and retention rate. A power analysis for an appropriate sample size would be warranted for a larger multi-year study. Despite the small sample size, we observed a very large difference between mean baseline and exit SKILLD scores relative to the pooled standard deviation, resulting in a large learning effect among clients. Study attrition was acceptable with 75% of recruited coaches retained and 69.2% of recruited clients retained. While thirteen workers with diabetes were recruited, only nine completed the program in full. Reasons for dropping included time, interest in the program, and, justifiably, the drop of one English-Spanish coach which caused the re-assignment of three Spanish-speaking clients between the two English-Spanish coaches. Even with new coach assignments, the three clients were able to reestablish relationships with new coaches and finish out the program.

### New Contribution to the Literature

Lessons learned from this pilot are pivotal for clinical care of agricultural workers with diabetes—especially those in health deserts. Training and coaching programs for hard-to-reach agricultural workers must be culturally, linguistically, and literacy appropriate for both coaches and clients. Future programs must be feasible and sustainable after research support and resources are no longer readily available. Future programs must focus on empowering community members as active participants in research and outreach initiatives, interventions, and applicable results. As researchers, we also have to capitalize on technological advances and persisting ‘new-normals’ from isolation and social distancing guidelines due to the pandemic—this includes virtual: gatherings, surveys, focus groups, consent signatures, telehealth appointments, among others in order to reach medically underserved rural communities. Last, programs must focus on dismantling common systemic barriers to health and intentionally empathizing with lived-experiences of agricultural workers and their families living in rural regions.
